# Osimertinib-induced biventricular cardiomyopathy with abnormal cardiac MRI findings: a case report

**DOI:** 10.1186/s40959-023-00190-1

**Published:** 2023-10-31

**Authors:** Karishma Patel, Kristie Y. Hsu, Kevin Lou, Krishan Soni, Yoo Jin Lee, Claire K. Mulvey, Alan H. Baik

**Affiliations:** 1 Department of Medicine, University of California, San Francisco, CA USA; 2 Department of Radiology, University of California, San Francisco, CA USA; 3 Division of Cardiology, University of California, San Francisco, CA USA; 4 Division of Oncology, University of California, San Francisco, CA USA; 5 Department of Medicine, Division of Cardiology, Section of Cardio-Oncology and Immunology, University of California, San Francisco, CA USA

**Keywords:** Osimertinib, Cardio-oncology, Cardiomyopathy, Pericardial effusion, Tamponade

## Abstract

**Background:**

Osimertinib is a third-generation epidermal growth factor receptor (EGFR) inhibitor that is currently the first-line treatment for metastatic EGFR-mutated non-small-cell lung cancer (NSCLC) due to its favorable efficacy and tolerability profile compared to previous generations of EGFR inhibitors. However, it can cause uncommon, yet serious, cardiovascular adverse effects.

**Case Presentation:**

We present the case of a 63-year-old man with EGFR-mutated NSCLC treated with osimertinib who developed new-onset non-ischemic cardiomyopathy with biventricular dysfunction and heart failure in the context of an enlarging pericardial effusion. For the first time, we demonstrate cardiac MR imaging findings associated with osimertinib-associated cardiomyopathy, including focal late gadolinium enhancement and myocardial edema. The patient’s biventricular function normalized after initiation of goal-directed medical therapy for heart failure and holding osimertinib. The patient was subsequently started on afatinib, a second-generation epidermal growth factor receptor-tyrosine kinase inhibitor (EGFR-TKI), without recurrence of cardiomyopathy.

**Conclusions:**

This case highlights the need to better understand osimertinib-induced cardiotoxicity and strategies to optimize oncologic care in patients who develop severe cardiac toxicities from cancer therapy. It further underlines the importance of specialized multidisciplinary care of cancer patients who develop cardiotoxicities to optimize their oncologic outcomes.

## Background

Osimertinib is a third-generation, irreversible EGFR inhibitor that selectively inhibits both EGFR TKI-sensitizing mutations, including EGFR-L858R and T790M mutations. Clinical trials have demonstrated superior efficacy and tolerability compared to first- and second-generation EGFR inhibitors. Therefore, osimertinib is the preferred first-line treatment of advanced EGFR-mutated non-small-cell lung cancer (NSCLC). Retrospective studies of the U.S. Food and Drug Administration Adverse Events Reporting System have shown that osimertinib is associated with cardiac toxicities, including heart failure, atrial fibrillation, and QT prolongation [1]. Up to 6.1% of patients treated with osimertinib have been reported to experience cardiac toxicities, compared to 2.1% of patients treated with earlier-generation EGFR-TKI inhibitors [2]. The mechanisms of osimertinib-induced cardiotoxicity are not well understood, though might be mediated by partial inhibition of HER2, additional off-target effects, and overlap with other risk factors for cardiovascular disease. Though osimertinib-associated cardiac adverse effects have previously been reported, the evaluation and management of severe cardiac toxicities are not well-established. We report a case of severe, acute biventricular dysfunction in a patient treated with osimertinib, and cardiac MR imaging findings consistent with non-ischemic cardiomyopathy. Although uncommon, the evaluation of osimertinib-induced cardiotoxicity is an important issue in clinical practice due to the frequency of use of osimertinib in the treatment of NSCLC.

## Case presentation

A 63-year-old man with a history of prior light tobacco use, hypertension, and type 2 diabetes mellitus presented with several months of progressive fatigue, weakness, and shortness of breath. CT chest demonstrated a left suprahilar mass, moderate left-sided pleural effusion, and numerous pulmonary, pleural, hepatic, and mediastinal metastatic lesions. Left upper lobe endobronchial ultrasound biopsy confirmed non-small-cell lung adenocarcinoma that was positive for an EGFR-L858R mutation. PET/CT demonstrated ~ 2 cm, PET-negative circumferential pericardial effusion. Pre-treatment transthoracic echocardiogram (TTE) demonstrated normal left ventricular ejection fraction (LVEF 65%) and grade I diastolic dysfunction, as well as a 1.5 cm pericardial effusion with no evidence of tamponade. He was started on osimertinib 80 mg daily. Four months later, follow-up PET/CT demonstrated reduction in overall disease burden as well as a stable circumferential pericardial effusion.

The patient had a repeat transthoracic echocardiogram (TTE) after three months of osimertinib treatment that demonstrated normal left ventricular size and function (LVEF 65%) with normal right ventricle function, and a moderate-large circumferential pericardial effusion. The patient had no signs or symptoms of tamponade. Due to persistence of the large pericardial effusion, which was presumed to be malignant, elective pericardiocentesis was discussed to avoid life-threatening tamponade. However, the patient deferred due to absence of symptoms or evidence of elevated right-sided filling pressures on exam at the time.

The patient was referred to cardio-oncology for a second opinion on management of the pericardial effusion. He denied angina, shortness of breath, dyspnea on exertion, dizziness, weight gain, or lower extremity edema. His blood pressure, heart rate, and oxygen saturation were normal. On cardiac exam, he had normal rhythm, distant heart sounds, normal S1 and S2, and no murmurs, rubs, or gallops. Jugular venous pressure (JVP) was less than 6 cmH_2_O and pulsus was less than 10 mmHg. Repeat TTE after four months on osimertinib demonstrated normal left and right ventricular function with normal LVEF (55–60%), large free-flowing pericardial effusion (measuring up to 2 cm), and exaggerated respiratory variation across the tricuspid valve inflow consistent with early tamponade. Semi-urgent pericardial window was recommended due to the risk of progression to tamponade. However, the patient declined intervention as he remained asymptomatic. Osimeritinib was continued due to absence of LV dysfunction.

**Fig. 1:**
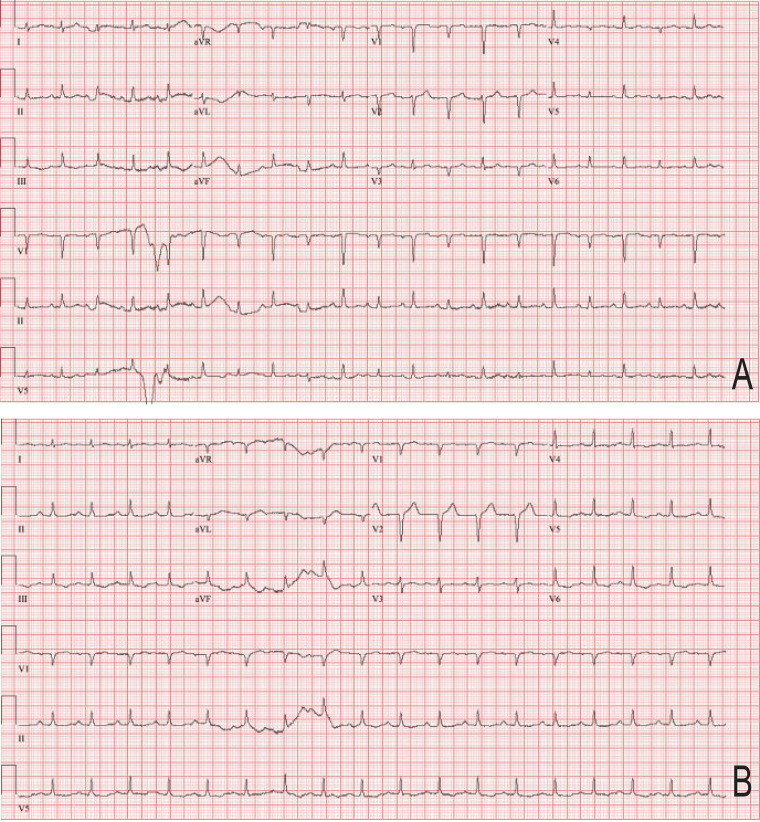
Electrocardiogram (ECG) prior to **(A)** and after **(B)** pericardiocentesis. **(A)** Initial presenting ECG with electrical alternans, in which the direction of electrical activity flips beat-to-beat in lead V3. **(B)** ECG after pericardiocentesis with resolution of electrical alternans

**Fig. 2:**
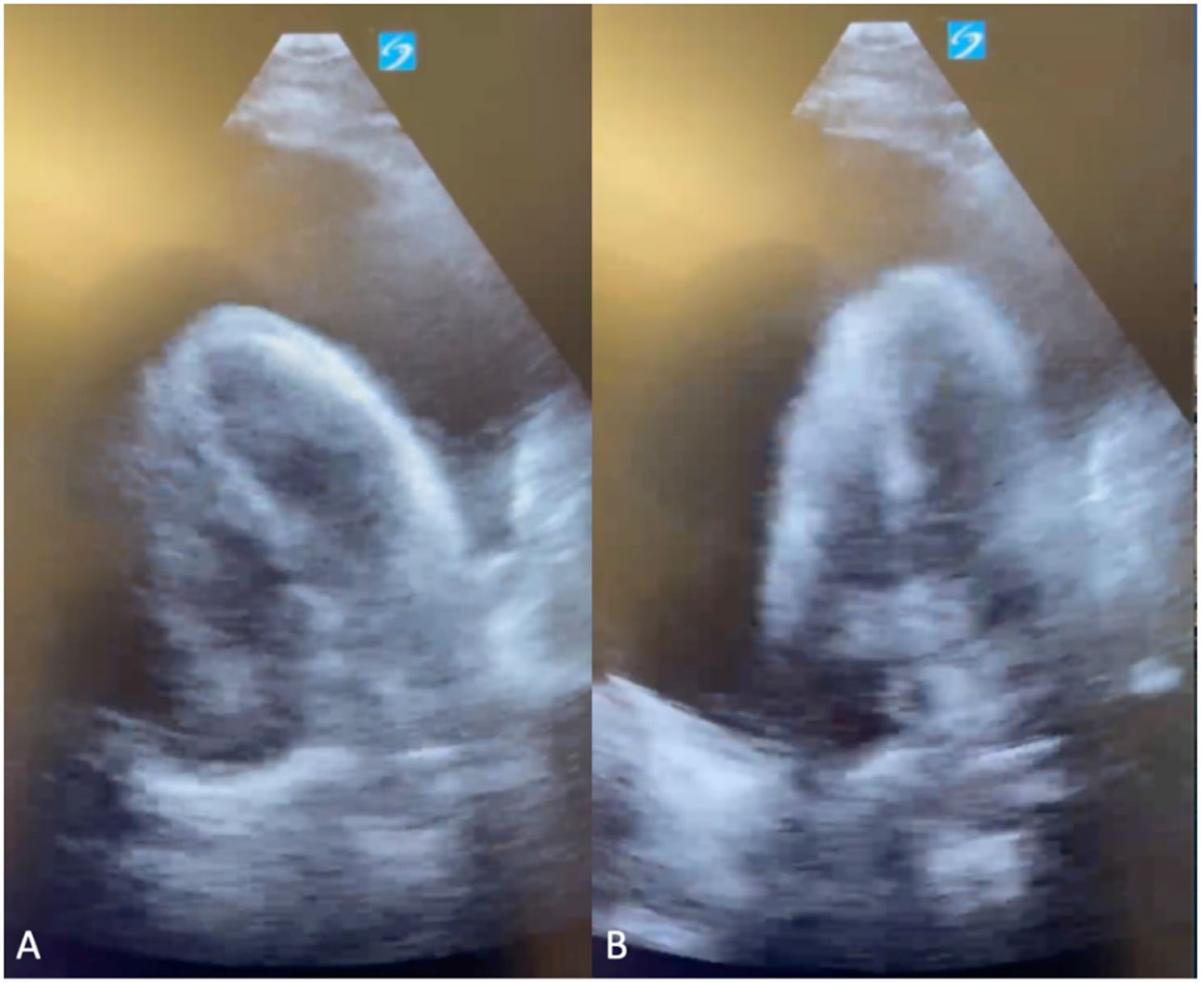
Point-of-care ultrasonography (POCUS) of large pericardial effusion. **(A)** POCUS demonstrates large pericardial effusion. **(B)** With ultrasound probe in same position as 1A, demonstrated swinging of heart within pericardial effusion

Four weeks later, the patient reported rapidly progressive weight gain and abdominal distention despite reduced oral intake and worsening dyspnea on exertion. He presented to his local emergency department (ED). Diminished breath sounds at the right lung base were noted on exam. ECG demonstrated sinus tachycardia. CT chest demonstrated a large pericardial effusion and moderate left pleural effusion. He underwent left-sided thoracentesis with removal of 850 cc pleural fluid and was advised to follow up with his outpatient cardio-oncologist regarding the pericardial effusion. Over the next three days, he experienced progressive shortness of breath and weight gain. He was advised to present to the ED emergently due to concern for cardiac tamponade. On exam, he was afebrile, tachycardic to 125 bpm, normotensive (BP 129/95 mmHg), and mildly hypoxic, requiring 2 L/min supplemental oxygen. Cardiac exam was notable for an elevated JVP at 14 cm H_2_O. Labs were significant for mild leukocytosis (WBC 10.7 × 10E9), C-reactive protein of 59.3 mg/L and sedimentation rate of 31 mm/hr. ECG demonstrated electrical alternans (Fig. 1A). Point-of-care ultrasonography (POCUS) demonstrated a large pericardial effusion (Fig. 2) with beat-to-beat swinging of the heart within the effusion. Due to clinical tamponade, he underwent emergent pericardiocentesis with removal of 1.1 L serosanguinous fluid and pericardial drain placement. In discussion with oncology, osimertinib was held in the emergent setting.

Repeat ECG following pericardiocentesis demonstrated sinus tachycardia with resolution of electrical alternans and without evidence of ischemia (Fig. 1B). The following day, the patient reported new-onset orthopnea. On exam, his BP was 90/68, HR 125 bpm, and JVP was elevated to 13 cmH_2_O. The pulsus was less than 10 mmHg. Furosemide was initiated to manage intravascular volume overload. After clamping of the pericardial drain, limited TTE showed a small to moderate pericardial effusion and newly reduced biventricular function (LVEF 30–35%) with akinesis of the mid-left ventricle, and anteroseptal wall motion abnormality. Initial troponin was 1.4 ug/L and peaked at 2.2 ug/L six hours later. Brain natriuretic peptide was 666 pg/mL. The patient underwent coronary angiography which showed mild, non-obstructive coronary artery disease and an elevated LVEDP of 17 mmHg.

The patient underwent minimally invasive pericardial window. Cytology results of the pericardial fluid demonstrated slightly dispersed and small cohesive clusters of neoplastic cells with intracytoplasmic vacuoles, enlarged nuclei and prominent nucleoli, with TTF1 positive and Napsin-A positive immunohistochemical stains, consistent with primary lung adenocarcinoma. Fluid analysis demonstrated hazy, light yellow liquid with negative bacterial culture (pH 7.33, protein 2.8 g/dL, lactate dehydrogenase (LDH) 137 U/L, bilirubin 2.8 mg/dL, triglycerides 39 mg/dL, hematocrit 13%, WBC 1.775 × 10E9, RBC 0.88 × 10E9).

**Fig. 3:**
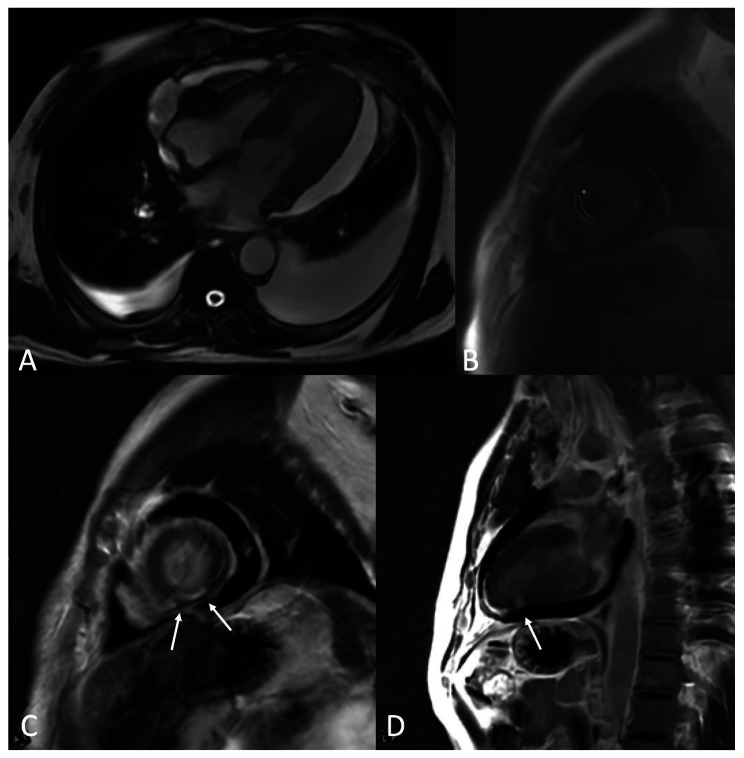
Cardiac MRI demonstrating myocardial edema and late gadolinium enhancement. **(A)** Circumferential pericardial effusion and bilateral pleural effusions. **(B)** Elevated T2 mapping value of 62.8 ms in the septum demonstrates the presence of myocardial edema. **(C, D)** Mid-wall pattern of late gadolinium enhancement in the mid-inferior left ventricular wall is consistent with a non-ischemic type cardiomyopathy as may be seen in acute myocarditis. The study was partly limited by motion artifact due to patient’s difficulty holding breath likely secondary to bilateral pleural effusions and pericardial effusion

Given the absence of obstructive coronary disease, osimertinib-induced cardiomyopathy was suspected. For management of heart failure with reduced ejection fraction (HFrEF), the patient was initiated on empagliflozin, spironolactone, and metoprolol. Due to low blood pressures, further up-titration of goal-directed medical therapy was limited. The patient had a heart rhythm monitor that showed normal sinus rhythm without evidence of arrhythmia. Cardiac MRI seven weeks after the initial diagnosis of cardiomyopathy demonstrated LVEF of 45%, mid-wall pattern late gadolinium enhancement, and elevated T2 mapping values at the mid-inferior left ventricular wall reflecting non-ischemic cardiomyopathy with associated myocardial edema (Fig. 3). Interval TTE three months after discontinuation of osimertinib showed normalization of the LV ejection fraction (65%) and trace pericardial effusion. The patient denied heart failure symptoms and was euvolemic on exam.

After a follow up PET/CT scan showed increased metabolic activity in several lymph nodes, the patient was started on afatinib 40 mg daily, a second-generation EGFR-TKI. Follow up PET/CT scan six weeks after initiating afatinib demonstrated stable to improved hypermetabolic disease, consistent with treatment response. Follow up TTE six months after initiating afatinib showed normal LV function and a stable trace pericardial effusion.

## Discussion and conclusions

We present a case of acute, severe biventricular cardiomyopathy due to osimeritinib in a patient with metastatic lung adenocarcinoma and malignant pericardial tamponade. Though there are prior case reports of cardiotoxicity related to osimertinib [2–6], this case is unique for several reasons: (1) we describe a case of suspected myocardial toxicity, leading to severe biventricular cardiomyopathy, (2) we present abnormal cardiac MRI findings of mid-wall pattern late gadolinium enhancement and elevated T2 mapping values of the left ventricular wall consistent with non-ischemic cardiomyopathy with associated myocardial edema, which have not previously been described in cases of osimertinib-induced cardiomyopathy, and (3) we describe successful treatment with a second-generation EGFR TKI without recurrence of cardiomyopathy or pericardial effusion following discontinuation of osimertinib. This case also underlines the importance of risk and benefit discussions with patients who are reluctant to undergo elective pericardial drainage for presumed malignant enlarging pericardial effusions in order to prevent life-threatening tamponade.

The initial differential for the patient’s new-onset, non-ischemic heart failure was broad, including ischemic cardiomyopathy, Takotsubo-variant cardiomyopathy, tachycardia-mediated cardiomyopathy, osimertinib-induced cardiomyopathy, and autoimmune or structural etiologies. Our patient’s underlying cardiac risk factors included prior tobacco use, South Asian descent, hypertension, and diabetes mellitus. He had no history of viral infection and no signs or symptoms of autoimmune or endocrine etiologies. He denied a history of any alcohol or illicit substance use. Ultimately, osimertinib was determined to be the likely culprit of the cardiomyopathy due to the temporal development of heart failure, negative workup of other etiologies, and recovery of ejection fraction following discontinuation of osimertinib. Cardiac MRI following the diagnosis of cardiomyopathy demonstrated LVEF of 45%, and focal late gadolinium enhancement and elevated T2 mapping values involving the mid-inferior left ventricular wall. Repeat TTE completed three months following osimertinib discontinuation showed normalization of ejection fraction.

While the underlying mechanisms of osimertinib-induced cardiac toxicities are currently not known, the lower frequency of adverse cardiac events seen with older-generation inhibitors suggests that inhibition of EGFR itself may not entirely mediate these toxicities. Notably, HER2 (EGFR2) and EGFR are in the same family of tyrosine kinase receptors. HER2 targeting therapies also have a risk of cardiotoxicity due to inhibition of HER2, which supports cardiac function and physiologic response to cardiac stress through involvement in cell survival pathways [7]. Pre-clinical studies suggest that osimertinib partially inhibits HER2, which might contribute to its cardiotoxic effects [7]. Additional studies are needed to determine which other off-target(s) might also contribute, particularly considering osimertinib’s cysteine-reactive warhead which might engage reactive thiols on many potential off-target proteins. Further structural optimization of T790M-targeting EGFR inhibitors, such as osimertinib and fourth generation inhibitors like BLU-945, or application of emerging allosteric EGFR inhibitors might be needed to simultaneously address the clinical challenges of resistance mutations and cardiotoxic side effects. Furthermore, prior studies suggest that patients with pre-existing cardiac risk factors are at higher risk of developing cardiotoxicity due to osimertinib [7].

Notably, this patient developed a worsening malignant pericardial effusion despite osimeritinib treatment and evidence of overall reduction in cancer burden. One possibility is that the penetration of osimertinib into effusions might be low. A retrospective study demonstrated that in patients with NSCLC with EGFR mutations, those with malignant effusions (pericardial, abdominal, and pleural) had significantly shorter progression-free survival compared to patients without effusions [8]. In contrast, Masago et al. showed that erlotinib drug concentrations increase in pleural effusions with repeated doses [9]. Additional possibilities include direct pericardial toxicity of osimertinib or accelerated progression of malignant effusions in patients treated with osimertinib. Therefore, more research is needed to better understand the pharmacokinetics, pharmacodynamics, and possible pericardial toxicities of osimeritinib in the context of malignant effusions.

To our knowledge, this is the first report of abnormal cardiac MRI findings in a patient with osimertinib-induced cardiomyopathy. Here, cardiac MRI demonstrated pericardial effusion and bilateral pleural effusions, focal myocardial edema, and late gadolinium enhancement (Fig. 3A-D). Cardiac MRI plays a valuable role in the diagnosis of non-ischemic cardiomyopathy [10], including osimertinib-induced cardiomyopathy, as demonstrated in this case. Non-ischemic cardiomyopathy can be identified from the pattern of myocardial scar seen as late gadolinium enhancement. Additional information that can be obtained from cardiac MRI include the presence of myocardial edema using T2 weighted imaging, presence of early or global fibrosis using T1 weighted imaging, decreased systolic function, and abnormal myocardial motion from cine imaging, in addition to pericardial abnormalities including enhancement, thickening, or local adhesion [10]. Repeat cardiac MRI can be considered to monitor for resolution of abnormal findings associated with osimertinib-induced cardiotoxicity.

Our patient was initiated on goal-directed medical therapy for HFrEF, including empagliflozin, spironolactone, losartan, and metoprolol succinate. Further optimization was limited due to low blood pressures and side effects. Ejection fraction measurement three months after stopping osimertinib was normal, further supporting that his cardiomyopathy was likely secondary to osimertinib toxicity, consistent with prior reports that osimertinib-associated cardioymyopathy is reversible with drug cessation [11]. Formal guidelines regarding management of EGFR inhibitor associated cardiac toxicities are currently lacking.

There are currently no established guidelines regarding treatment modification in the setting of osimertinib-induced cardiomyopathy, although cases of osimertinib re-challenge and/or dose reduction have also been reported [5, 6, 12, 13]. We reasoned that osimertinib-induced cardiomyopathy might involve an off-target that was non-overlapping with earlier generation EGFR TKIs and therefore favored EGFR TKI substitution as a strategy to prevent re-emergence of adverse cardiac side-effects. As a second-generation EGFR TKI, afatinib has not been studied specifically in patients with heart failure but clinical trials have reported low incidence of cardiotoxicity [14]. The patient was started on afatinib 40 mg daily. PET/CT five weeks after starting afatinib demonstrated cancer response, and TTE six months after initiating afatinib showed normal LV function and a stable trace pericardial effusion.

The cardiotoxic effects of osimertinib include non-ischemic cardiomyopathy and must be considered in patients with NSCLC, especially in patients with underlying risk factors for cardiac disease. MRI may demonstrate focal late gadolinium enhancement and elevated T2 mapping values at the left ventricular wall reflective of non-ischemic cardiomyopathy with associated myocardial edema. Following discontinuation of osimertinib, cardiac function may return to baseline and patients may be successfully treated with second-generation EGFR TKI without recurrence of cardiomyopathy.

## Data Availability

Data sharing is not applicable to this article as no datasets were generated or analysed during the current study.

## List of abbreviations

EGFR: Epidermal growth factor receptor
TKI: Tyrosine kinase inhibitor
NSCLC: Non-small-cell lung cancer
TTE: Transthoracic echocardiogram
LVEF: Left ventricular ejection fraction
JVP: Jugular venous pressure
ED: Emergency department
POCUS: Point-of-care ultrasonography
HFrEF: Heart failure with reduced ejection fraction
ECG: Electrocardiogram
