# Correction: Osimertinib-induced biventricular cardiomyopathy with abnormal cardiac MRI findings: a case report

**DOI:** 10.1186/s40959-023-00195-w

**Published:** 2023-11-29

**Authors:** Karishma Patel, Kristie Y. Hsu, Kevin Lou, Krishan Soni, Yoo Jin Lee, Claire K. Mulvey, Alan H. Baik

**Affiliations:** 1 Department of Medicine, University of California, San Francisco, CA USA; 2 Department of Radiology, University of California, San Francisco, CA USA; 3 Division of Cardiology, University of California, San Francisco, CA USA; 4 Division of Oncology, University of California, San Francisco, CA USA; 5 Department of Medicine, Division of Cardiology, Section of Cardio- Oncology and Immunology, University of California, San Francisco, CA USA

**Correction: Cardio-Oncol 9, 38 (2023)**

**https://doi.org/10.1186/s40959-023-00190-1**

Following publication of the original article [1], the authors identified an error in Fig. 1, in which Fig. 1A and B were reversed. The correct figure is given below.

**Fig. 1:**
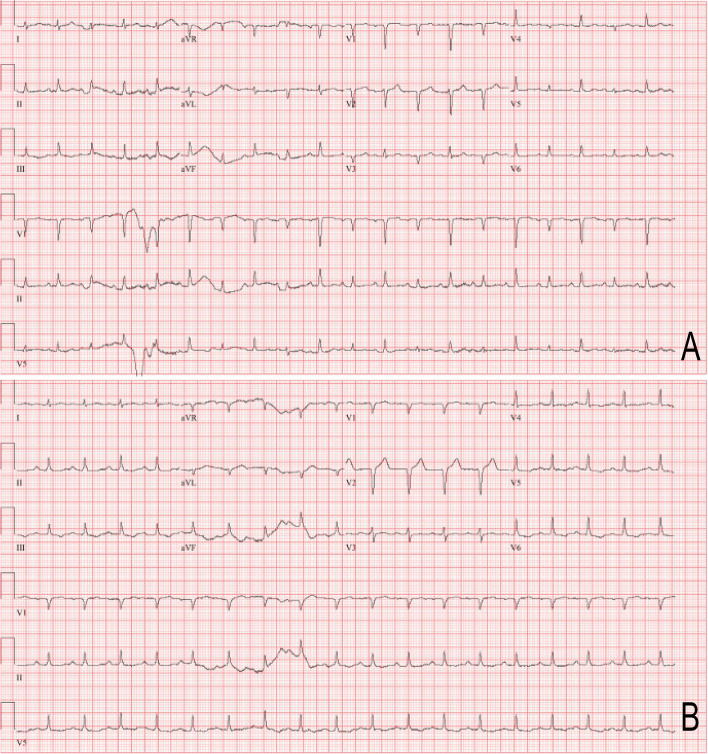
Electrocardiogram (ECG) prior to (**A**) and after (**B**) pericardiocentesis. (**A**) Initial presenting ECG with electrical alternans, in which the direction of electrical activity flips beat-to-beat in lead V3. (**B**) ECG after pericardiocentesis with resolution of electrical alternans

The original article [1] has been updated.
